# Inducible Nitric Oxide Regulates Na-Glucose Co-Transport in a Spontaneous SAMP1/YitFc Mouse Model of Chronic Ileitis

**DOI:** 10.3390/nu12103116

**Published:** 2020-10-13

**Authors:** Balasubramanian Palaniappan, Shanmuga Sundaram, Subha Arthur, Sheuli Afroz, Uma Sundaram

**Affiliations:** Department of Clinical and Translational Sciences, Joan C. Edwards School of Medicine, Marshall University, Huntington, WV 25701, USA; palaniappan@marshall.edu (B.P.); sundaram1@live.marshall.edu (S.S.); arthursu@marshall.edu (S.A.); afroz@marshall.edu (S.A.)

**Keywords:** SAMP1/YitFc, SGLT1, inflammatory bowel disease, inducible nitric oxide, L-NIL

## Abstract

In mammalian small intestine, glucose is primarily absorbed via Na-dependent glucose co-transporter (SGLT1) on the brush border membrane (BBM) of absorptive villus cells. Malabsorption of nutrients (e.g., glucose) leads to malnutrition, a common symptom of inflammatory bowel disease (IBD), where the mucosa is characterized by chronic inflammation. Inducible nitric oxide (iNO) is known to be elevated in IBD mucosa. SAMP1/YitFc (SAMP1) mouse is a spontaneous model of chronic ileitis that develops lesions in its terminal ileum, very similar to human IBD. How SGLT1 may be affected in SAMP1 model of chronic ileitis is unknown. Ten-week-old SAMP1 mice with AKR mice as control were treated with N6-(1-iminoethyl)-L-lysine dihydrochloride (L-NIL) to inhibit iNO production. Intracellular NO levels were found to be increased in villus cells from SAMP1 mice. Moreover, SGLT1 and Na^+/^K^+^-ATPase activities and BBM SGLT1 expression were significantly decreased. However, L-NIL treatment reduced the intracellular iNO production, and reversed both downregulated SGLT1 and Na^+^/K^+^-ATPase activities in SAMP1 mice. Inhibition of iNO by L-NIL treatment also significantly reversed the BBM SGLT1 protein expression in SAMP1 mice. L-NIL reversed the inflammation mediated downregulation of SGLT1 activity by restoring the BBM SGLT1 expression. Thus, regulation of SGLT1 in chronic ileitis is likely mediated by iNO.

## 1. Introduction

Na-glucose co-transport (SGLT1), found in the brush border membrane (BBM) of the absorptive villus cells is the most abundant Na-dependent nutrient transporter present in the mammalian small intestine. Since it is a secondary active transporter, Na^+^/K^+^-ATPase present in the basolateral membrane (BLM) of villus cells provides a favorable transcellular Na gradient required for its optimal activity [1]. Therefore, SGLT1’s activity can be regulated in the villus cells at the level of BBM co-transporter and/or at the level of Na-extruding capacity of the cell. SGLT1 is not only an important absorptive mechanism for Na in the small intestine, but also plays a significant role in the absorption of glucose, the most abundant dietary nutrient [2]. In fact, preserved function of SGLT1 is the basis for the most efficacious and affordable treatment, specifically oral rehydration solution, for the number one cause of infant mortality in the developing countries, namely diarrheal diseases.

Inflammatory Bowel Disease (IBD; Crohn’s diseases and Ulcerative Colitis) is a universal disease in the 21st century, with the highest prevalence recorded in Northern America and Europe [3]. Approximately, 3.1 million or 1.3% of U.S. adults (aged ≥ 45 years) have been diagnosed with IBD [4]. IBD in humans has been well studied in recent years, and is caused by several factors including host immune system, genetic predisposition, intestinal microbiome and environmental factors [5]. Of all these influences, host immune mediators are the major contributors of the severity and progression of intestinal mucosal inflammation in patients with IBD. Impairment of NaCl absorption and reduction of nutrient absorption resulting in malabsorption, diarrhea, malnutrition and weight loss are the most important sequelae interfered by different immune-inflammatory meditators such as eicosanoids (prostaglandins, leukotrienes, thromboxanes), nitric oxide, neuropeptides, and cytokines (IL-1β, TNFα, IFN-γ) in IBD [6,7]. Our previous studies have demonstrated unique changes in various nutrient absorptive processes such as Na-dependent glucose, amino acids such as glutamine and alanine, dipeptides and bile acids, in the chronically inflamed rabbit intestine [8,9,10,11]. These alterations directly affect the co-transporters in BBM and are secondary to the Na-extruding capacity of the enterocytes at cellular level.

Nitric oxide (NO) is a signaling molecule and an important immune inflammatory mediator that is well-known to play a key role in the pathogenesis of inflammation. Inducible NO (iNO) produced by iNO synthase (iNOS) plays a variety of etiological roles in human chronic inflammatory diseases, which was well established in early 1990s [12]. Furthermore, inhibition of iNOS reduced the inflammation, thus demonstrating the involvement of iNO as an inflammatory mediator during inflammation [13,14,15,16]. Various studies have also shown that during chronic inflammation, selective inhibition of iNOS ameliorates tissue damage [17,18,19,20].

The SAMP1/YitFc mouse model of ileitis, resembling human Crohn’s disease (CD), has been found to be well suited for investigating the pathogenesis and treatment of chronic enteritis [21,22,23]. SAMP1/YitFc mouse model is known to spontaneously develop chronic ileitis without any genetic, chemical, or immunological manipulation. In addition, SAMP1/YitFc mouse has been demonstrated to have remarkable similarities to the human chronic enteritis with respect to the location of inflammation, histologic features, and most importantly, its response to conventional therapies [21].

Although iNO is a well-known factor involved in human IBD as demonstrated by various studies, a big lacuna of the specific role of iNO in the regulation of Na-dependent nutrient transporters in chronic intestinal inflammation still exists. Given this background, the aim of the present study is to understand if and how BBM Na-glucose co-transport may be regulated by iNO in SAMP1/YitFc mice and to understand the functional and molecular mechanisms that may be involved in their regulation.

## 2. Materials and Methods

### 2.1. Animals and Treatment

Ten-week-old SAMP1/YitFcs mice obtained from The Jackson Laboratory, USA was used as a spontaneous model of chronic ileitis with AKR/J as the control strain. The animals were maintained in 12 h light/dark cycle and controlled room temperature with free access to water and food. To selectively inhibit iNO, the mice were treated intraperitoneally with N6-(1-iminoethyl)-L-lysine dihydrochloride (L-NIL; 0.1 mg/Kg) for 2 days to inhibit iNO production, while the control animals received sterile distilled water. All animal handling, treatments and euthanization were carried out according to the ethical considerations and protocols (IACUC code: 743) approved by Marshall University Institutional Animal Care and Use Committee (IACUC).

Intestinal villus cells were obtained from these mice either by Ca^++^ chelating technique as described before [24,25] or by scrapping the intestinal mucosa. BBM vesicles (BBMV) were prepared using divalent cation (Mg^2+^) chelation and differential centrifugation technique as previously reported [24,25]. BBMV were suspended either in an appropriate reaction buffer for uptake experiments or in an appropriate buffer for molecular studies.

### 2.2. Measurement of NO

Total NO levels were measured in villus cells using Griess reaction colorimetric assay (Cayman Chemicals, Ann Arbor, MI, USA) according to the manufacturer’s instruction. Briefly, previously frozen villus cells were lysed with phosphate buffered saline (PBS) by sonication and then centrifuged to obtain clear supernatants, which were then used for the assay. Nitrite and nitrate concentrations were then measured by a colorimetric assay with the nitrate standard curve, and the assayed samples were read at 540 nm.

### 2.3. Na-Glucose Co-Transport Uptake Studies in Intact Villus Cells and BBMV

Uptake studies in intact villus cells and BBMVs were performed by the rapid-filtration technique as described previously [24,25,26] with ^3^H-O-methyl glucose (OMG) as the substrate. OMG is a non-metabolisable glucose analog, so can be conveniently used as a marker to assess glucose transport by evaluating its uptake. Briefly, for intact villus cell uptake, 10 μL of villus cells were suspended in Na-free buffer containing 130 mM trimethyl ammonium chloride (TMACl), 4.7 mM KCl, 1 mM MgSO_4_, 1.25 mM CaCl_2_ and 20 mM Tris-HEPES (pH 7.4). The villus cells were then incubated in 90 μL of reaction medium that contained 130 mM NaCl, 4.7 mM KCl, 1 mM MgSO_4_, 1.25 mM CaCl_2_, 20 mM Tris-HEPES (pH 7.4 at room temperature), 10 μCi of ^3^H-OMG and 100 μM OMG in the presence or absence of 1 mM phlorizin. Uptakes were arrested at 2 min for intact villus cell uptakes or at 60 s for BBMV uptakes by mixing with ice-cold stop solution (Na-free buffer) containing 25 mM D-glucose. The mixture was filtered through specific filters (0.65 μm Millipore (HAWP) filters for intact cells and 0.45 μm Millipore (HAWP) filters for BBMV) and washed twice with 5 mL ice-cold stop solution. The filters were then dissolved in 4 mL scintillation fluid (Ecoscint A, National Diagnostics, Atlanta, GA, USA) overnight, and radioactivity was determined in a Beckman Coulter 6500 Beta Scintillation Counter.

### 2.4. Na^+^/K^+^-ATPase Enzyme Measurement

As previously described [27], Na^+^/K^+^-ATPase activity was measured as *P_i_* liberated in an equal amount of cellular homogenate prepared from isolated mice villus cells from different experimental conditions. Na^+^/K^+^-ATPase activity was defined as nanomoles of *P_i_* released per milligram protein per minute.

### 2.5. RT-qPCR Analysis

Total RNA from villus cells were extracted using RNeasy mini kit (74106; Qiagen, Germantown, MD, USA) followed by first strand cDNA synthesis with High capacity cDNA Reverse Transcription kit (4368814; Applied Biosystems, Foster City, CA, USA). Quantitative reverse transcription PCR experiments for mouse β-actin (Mm01205647 g1) and mouse specific SGLT1 (Mm00451210 m1) were performed using TaqMan^®^ Gene Expression Assays obtained from Applied Biosystems. Beta-actin RT-qPCR, which served as the endogenous control, was run along with the SGLT1 in similar experimental conditions (40 cycles: 95 °C for 15 s and 60 °C for 1 min). The data obtained with β-actin was used to normalize the expression levels of SGLT1 between individual samples. To ensure optimal efficiency and reproducibility of RT-qPCR, different dilutes of cDNA were tested and RT-qPCR experiments were performed at least three times using total RNA isolated from different sets of isolated villus cells.

### 2.6. Western Blot Analysis

Western blot analysis of cellular and BBM proteins were performed according to standard protocols. Protein extracts were solubilized in RIPA buffer (50 mM Tris-HCl pH 7.4, 1% Igepal, 150 mM NaCl, 1 mM EDTA, 1 mM PMSF, 1 mM Na_3_VO_4_, 1 mM NaF) with protease inhibitor cocktail (SAFC Biosciences, Lenexa, KS, USA) and was then mixed with sample buffer (100 mM Tris, 25% glycerol, 2% SDS, 0.01% bromophenol blue, 10% 2-ME, pH 6.8) and separated on a custom-made 8% poly acrylamide gel. The separated proteins were transferred to BioTrace PVDF membrane and probed with anti-SGLT1 antibody (07-1417, Millipore Sigma, Burlington, MA, USA) raised in rabbit. The primary antibody bound to the SGLT1 protein was detected with mouse anti-rabbit (sc-2357, Santa Cruz Biotechnology Inc., Dallas, TX, USA) horseradish peroxidase coupled secondary antibody. The resulting chemiluminescence was measured by autoradiography with an ECL Detection Reagent (GE Healthcare, Chicago, IL, USA) and the SGLT1 specific protein density was then quantitated via a densitometric scanner FluorChemTM instrument (Alpha Innotech, San Leandro, CA, USA).

### 2.7. Histology and Immunofluorescence Study

Segments of distal ileum were fixed in 10% (vol/vol) neutral-buffered formalin (Sigma Aldrich, St Louis, MO, USA) and embedded in paraffin. Deparaffinized (5 µm) sections were hydrated with graded ethanol and used for hematoxylin and eosin (H&E; Electron microscopy Science, Hatfield, PA, USA) staining and immunofluorescence. H&E stained images were captured using Moticam5 camera connected to a light microscope. For the immunofluorescence study of SGLT1, sections were incubated with 10 mM sodium citrate buffer, pH 6, at 95 °C for 10 min for antigen retrieval and washed three times with PBST (0.05% Tween-20 in phosphate buffered saline) as described previously [28]. Sections were then blocked with 2% bovine serum albumin for 1 h at room temperature. The sections were then incubated with anti-chicken SGLT1 primary antibody (custom antibody, Invitrogen Life Technologies) at a dilution of 1:250 for 1 h at room temperature followed by incubation with secondary antibody Alexa Fluor 488 goat anti-chicken (Cat No. A11039; Invitrogen Molecular Probes, Carlsbad, CA, USA) at 1:500 dilution for 1 h at room temperature. Unbound antibody was washed with PBST thrice, and the section was mounted with Fluoroshield mounting medium with 4, 9, 6-diamidino-2-phenylindole (DAPI) for nucleus staining (ab104139, Abcam, Cambridge, MA, USA). EVOS FL Cell imaging system was used to capture the images and quantified with AlphaView software version 3.4.0.0.

### 2.8. Protein Quantification

Proteins were quantified with the DCTM protein assay kit (Lowry’s method), according to manufacturer’s protocol (Bio-Rad, Berkeley, CA, USA) for all the uptake and molecular studies described in this study.

### 2.9. Statistics

In this study, results are expressed as means ± SE. The “n” number indicates experiments performed with cells isolated from different animals. For all the uptake experiments, each “n” also indicates uptakes performed as a triplicate. Student’s t-test was performed for statistical analysis with GraphPad Prism 7 (San Diego, CA, USA) software.

## 3. Results

**Figure 1:**
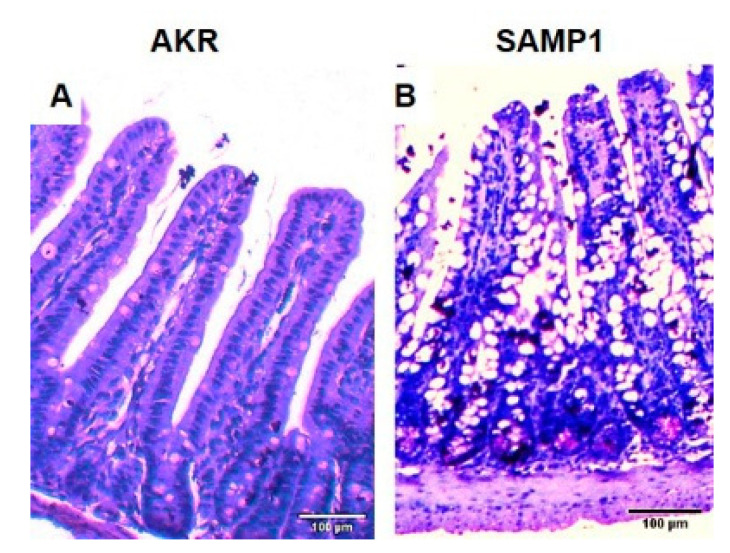
Representative example of cross section of the ileum of AKR and SAMP1 mouse stained with H&E. (**A**) AKR mouse intestine. (**B**) SAMP1 mouse intestine demonstrating crypt hypertrophy, villus blunting, and increased intra-epithelial lymphocytes characteristic of inflammatory bowel disease (IBD). Scale bar 100 µm.

Figure 1 is a representative example of cross section of the ileum of AKR and SAMP1 mouse. Hematoxylin and eosin stain was used to stain the sections. The AKR mouse ileum depicts the typical long villi, short crypts and minimal intraepithelial immunocytes. On the other hand, the chronically inflamed ileum of SAMP1 mouse demonstrates crypt hypertrophy, villus blunting, and increased intra-epithelial lymphocytes characteristic of IBD.

**Figure 2:**
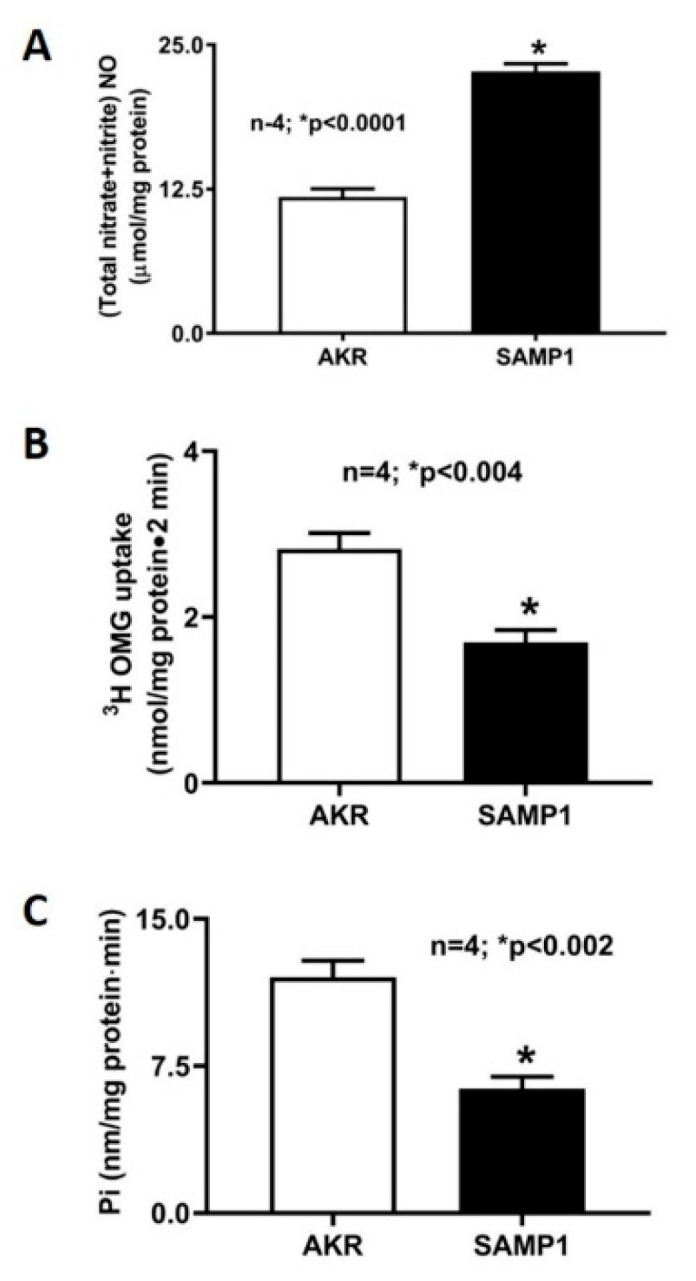
Physiological consequences of chronic intestinal inflammation in SAMP1 mouse. (**A**) Nitric oxide (NO): Mucosal levels of NO are significantly increased in SAMP1 mouse intestine. (**B**) Na-glucose co-transport (SGLT1): Na-dependent and phlorizin-sensitive ^3^H-OMG uptake is significantly decreased in villus cells from the SAMP1 intestine. (**C**) Na^+^/K^+^-ATPase: The activity of Na^+^/K^+^-ATPase, which provides the favorable Na gradient for SGLT1, is significantly diminished in SAMP1 mice intestine.

Figure 2 illustrates the pathophysiological consequences of chronic intestinal inflammation in SAMP1 mice. Mucosal levels of NO are significantly increased in SAMP1 mouse intestine (Figure 2A). More importantly, Na-glucose co-transport (SGLT1) activity is significantly decreased in villus cells from the SAMP1 intestine (Figure 2B). Na^+^/K^+^-ATPase, which provides the favorable Na gradient for SGLT1 in villus cells, is also significantly diminished in SAMP1 intestine (Figure 2C).

### 3.1. Effect of L-NIL on Intracellular NO Levels

**Figure 3:**
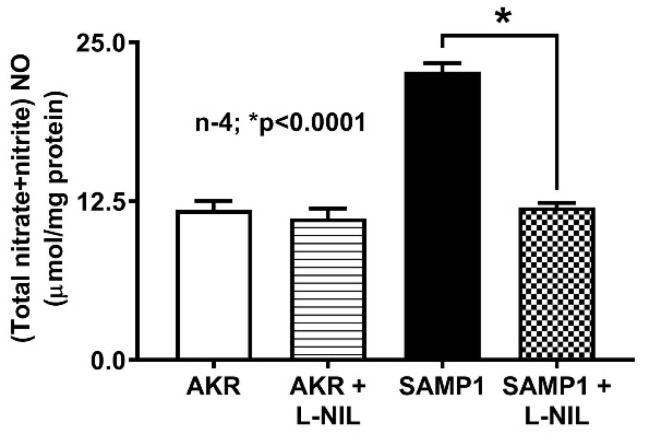
Effect of N6-(1-iminoethyl)-L-lysine dihydrochloride (L-NIL) on intracellular NO levels. NO level was significantly increased in ileal villus cells of SAMP1 mice compared to control AKR mice. L-NIL treatment reversed the stimulation of intracellular NO levels in SAMP1 mice, and had no effect on NO levels in villus cells from AKR mice.

As previously noted, iNO is known to be abundant in the chronically inflamed intestine and known to affect transport processes. Intracellular NO level was significantly increased in the villus cells from SAMP1 mice compared to that from AKR mice (Figure 3). However, this increased NO levels in SAMP1 mice was reversed back to normal levels by L-NIL treatment of SAMP1 mice. There was no change in NO levels in AKR mice treated with L-NIL (Figure 3).

### 3.2. Effect of L-NIL Treatment on Na-Glucose Co-Transport in Intact Villus Cells

**Figure 4:**
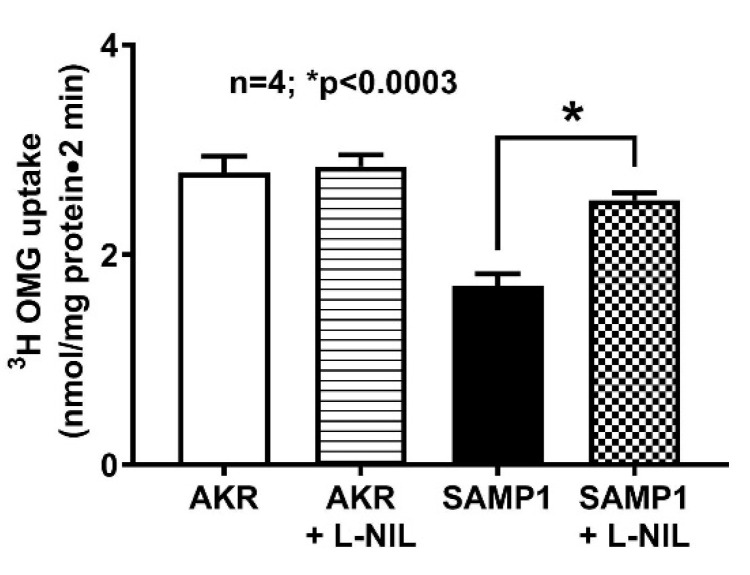
Effect of L-NIL treatment on Na-glucose co-transport in intact villus cells. Na-dependent ^3^H-OMG uptake that was significantly decreased during spontaneous ileitis in SAMP1 mice was reversed back to normal by L-NIL treatment. However, Na-glucose co-transport was unchanged in ileal villus cells isolated from AKR mice treated with L-NIL compared to the untreated control mice.

To determine the effect of iNO on SGLT1 activity during chronic intestinal inflammation, mice were administered with L-NIL. As shown in Figure 4, ^3^H-OMG uptake was significantly decreased in SAMP1 mice compared with control AKR mice (2.78 ± 0.16 nmol/mg protein•2 min in AKR; 1.71 ± 0.12 in SAMP1; n = 4, *p* < 0.0003). Moreover, L-NIL treatment reversed the inhibition of SGLT1 in SAMP1 mice (2.52 ± 0.07 nmol/mg protein•2 min in SAMP1 + L-NIL), but had no effect in AKR mice (2.84 ± 0.11 nmol/mg protein•2 min in AKR + L-NIL). These data demonstrated that iNO inhibits SGLT1 activity during inflammation, which was reversed by L-NIL treatment by inhibiting iNO production in ileal villus cells.

### 3.3. Effect of L-NIL on Na^+^/K^+^-ATPase Activity

**Figure 5:**
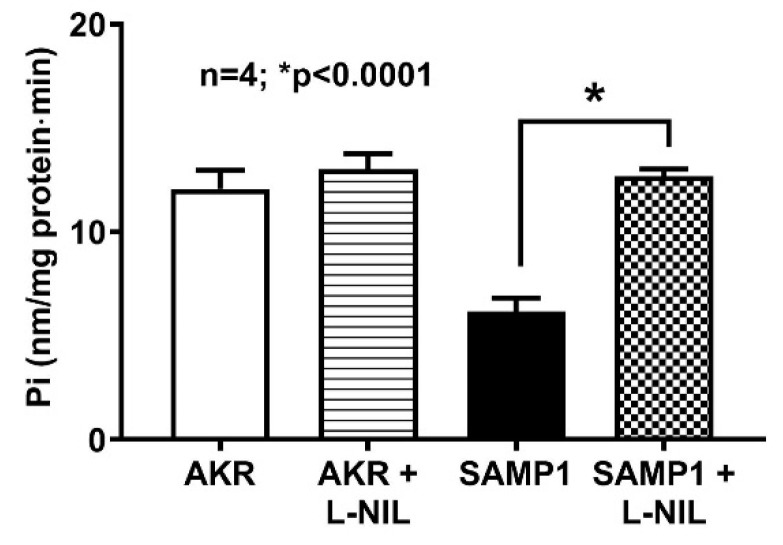
Effect of L-NIL treatment on Na^+^/K^+^-ATPase activity. Na^+^/K^+^-ATPase activity which was significantly reduced in SAMP1 mice intestinal villus cells was reversed back to normal levels by L-NIL treatment. L-NIL treatment did not change Na^+^/K^+^-ATPase activity in AKR mice intestinal villus cells.

Basolateral membrane Na^+^/K^+^-ATPase provides the favorable transcellular Na gradient required for an effective Na-dependent glucose absorption in villus cells. In this study, Na^+^/K^+^-ATPase activity was significantly inhibited in the villus cell homogenates from SAMP1 mice, indicating that Na^+^/K^+^-ATPase might partly be responsible for the reduced SGLT1 activity in the intact villus cells from SAMP1 mice. In SAMP1 mice treated with L-NIL, there was a reversal of Na^+^/K^+^-ATPase activity to normal levels (Figure 5; 12.1 ± 0.9 nmol/mg protein•min in AKR; 6.2 ± 0.6 in SAMP1; 13 ± 0.8 in AKR + L-NIL; 12.7 ± 0.4 in SAMP1 + L-NIL; n = 4, *p* < 0.0001). Therefore, iNO diminished both the Na driving gradient for SGLT1 in SAMP1 mice and indeed, diminished Na^+^/K^+^-ATPase activity.

### 3.4. Effect of L-NIL on Na-Glucose Co-Transport in the BBMV of Ileal Villus Cells

**Figure 6:**
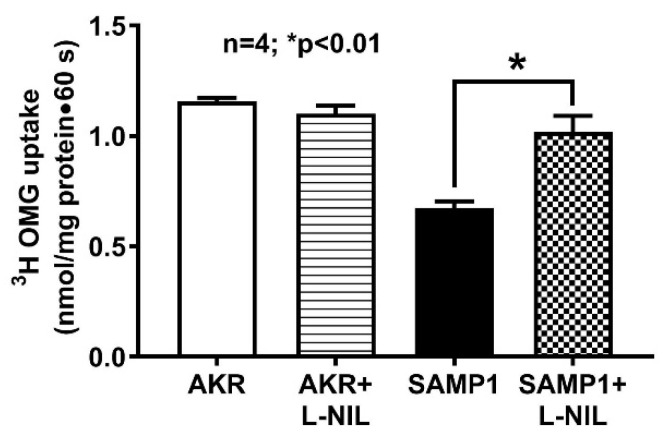
Effect of L-NIL treatment on Na-glucose co-transport in BBMV. Na-dependent ^3^H-OMG uptake was significantly decreased in ileal villus cell BBMV from SAMP1 mice. L-NIL treatment reversed the decreased BBM SGLT1 activity in SAMP1 mice to normal levels. Villus BBM Na-dependent glucose co-transport activity in villus cells from L-NIL treated and untreated AKR mice intestine remained unchanged.

To determine the direct effect of iNO on BBM Na-glucose co-transport, we performed ^3^H-OMG uptake experiments in BBMV prepared from villus cells isolated from treated and untreated AKR and SAMP1 mice. ^3^H-OMG uptake was significantly reduced in villus cell BBMV from SAMP1 mice compared to AKR mice. Moreover, L-NIL treatment completely reversed this reduced BBMV ^3^H-OMG uptake back to its normal levels (Figure 6; 1.16 ± 0.02 nmol/mg protein•1 min in AKR; 0.67 ± 0.03 in SAMP1; 1.1 ± 0.04 in AKR + L-NIL; 1.02 ± 0.08 in SAMP1 + L-NIL; n = 4, *p* < 0.01). L-NIL treatment did not affect SGLT1 activity in AKR mice.

### 3.5. SGLT1 mRNA Expressions by RT-qPCR

**Figure 7:**
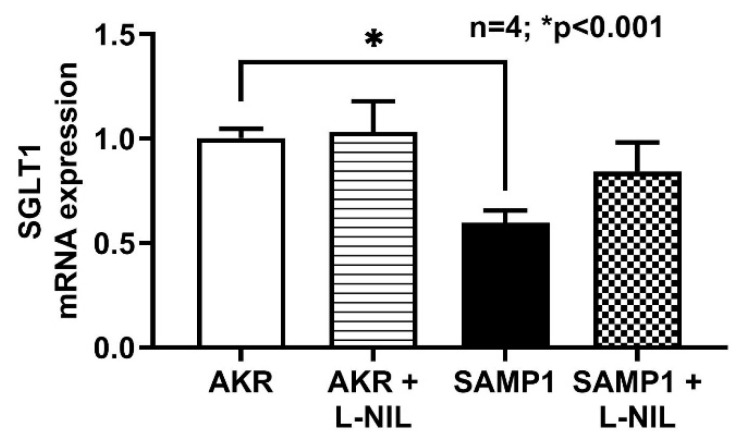
RT-qPCR analysis of SGLT1 mRNA expression. SGLT1 mRNA expression levels were significantly inhibited in SAMP1 mice villus cells compared with control AKR mice. L-NIL treatment reversed the inhibition of SGLT1 mRNA expression levels in SAMP1 mice to normal levels. However, SGLT1 mRNA expression levels remained unchanged between AKR mice untreated and treated with L-NIL.

SGLT1 mRNA expression was significantly reduced in SAMP1 mice villus cells compared to AKR. Inhibition of iNO by L-NIL reversed the inhibition of SGLT1 mRNA expression in SAMP1 mice villus cells. However, L-NIL did not alter the SGLT1 mRNA expression in AKR mice (Figure 7). This result reveals that iNO mediated inhibition of SGLT1 is likely through altered mRNA expression.

### 3.6. Quantitation of SGLT1 Protein by Western Blot Analysis

**Figure 8:**
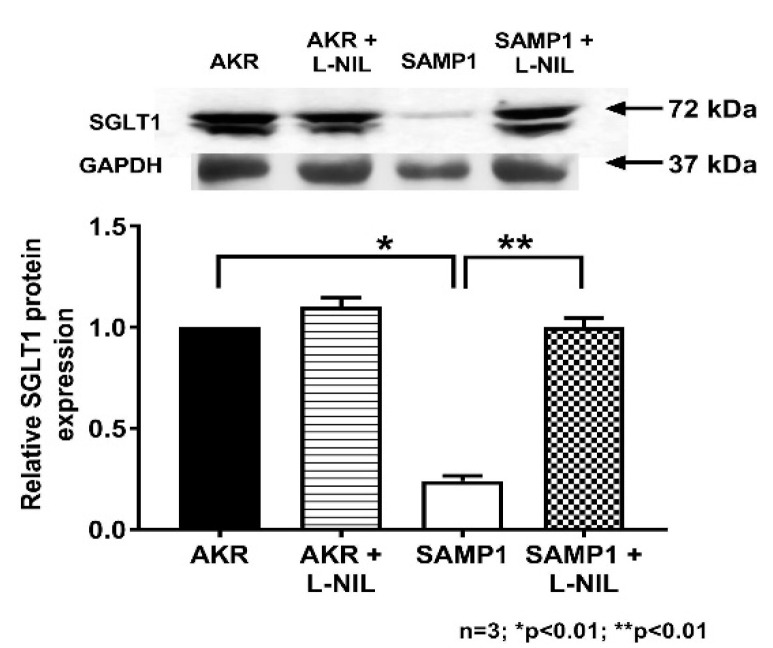
Effect of L-NIL on villus cell SGLT1 protein expression in SAMP1 mouse intestine. The panel on the top is the representative blot that shows that the villus cell SGLT1 expression in SAMP1 mice was significantly reduced, which was reversed back to normal levels by L-NIL treatment. SGLT1 levels remained unaltered by L-NIL treatment in AKR mice. Densitometric analysis as shown in the bottom panel confirmed these findings.

To determine the molecular mechanism of iNO mediated inhibition of SGLT1 in villus cells, Western blot studies were performed. First in cellular homogenates, SGLT1 protein levels in villus cells were quantitated as shown in Figure 8. Western blot studies revealed that SGLT1 protein level was significantly reduced in SAMP1 mice villus cells and this was restored to normal levels by L-NIL treatment (Figure 8). Densitometry analyses demonstrated that the iNO mediated inhibition of SGLT1 activity in SAMP1 mice villus cells was secondary to reduced co-transporter numbers.

**Figure 9:**
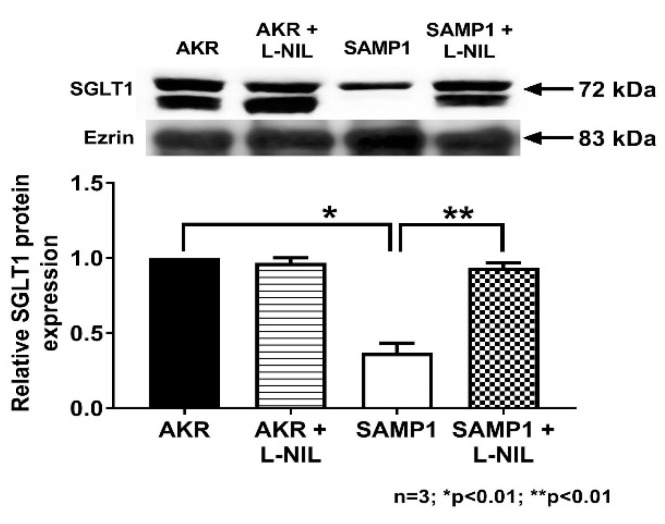
Effect of L-NIL on villus cell BBM SGLT1 protein expression during chronic ileitis. The panel on the top is the representative blot that shows that the BBM levels of SGLT1 expression in SAMP1 mice was significantly reduced, and was reversed back to normal levels by L-NIL treatment. BBM SGLT1 levels remained unaltered by L-NIL treatment in AKR mice. Densitometric analysis as shown in the bottom panel confirmed these findings.

SGLT1 is a BBM co-transporter requiring trafficking to the membrane. So to determine whether the decrease in SGLT1 by iNO in SAMP1 villus cells is secondary to a decrease in the synthesis and/or trafficking to the plasma membrane, BBM SGLT1 protein levels in villus cells were quantitated as shown in Figure 9. Western blot studies revealed that BBM SGLT1 protein level was significantly reduced in SAMP1 mice villus cell BBM, but was reversed back to normal levels by L-NIL treatment (Figure 9). Western blot densitometry analyses demonstrated that the inhibition of SGLT1 activity in SAMP1 mice by iNO was likely secondary to diminished BBM co-transporter numbers.

### 3.7. Immunohistochemistry of SGLT1 in SAMP1 Mice Intestine

**Figure 10:**
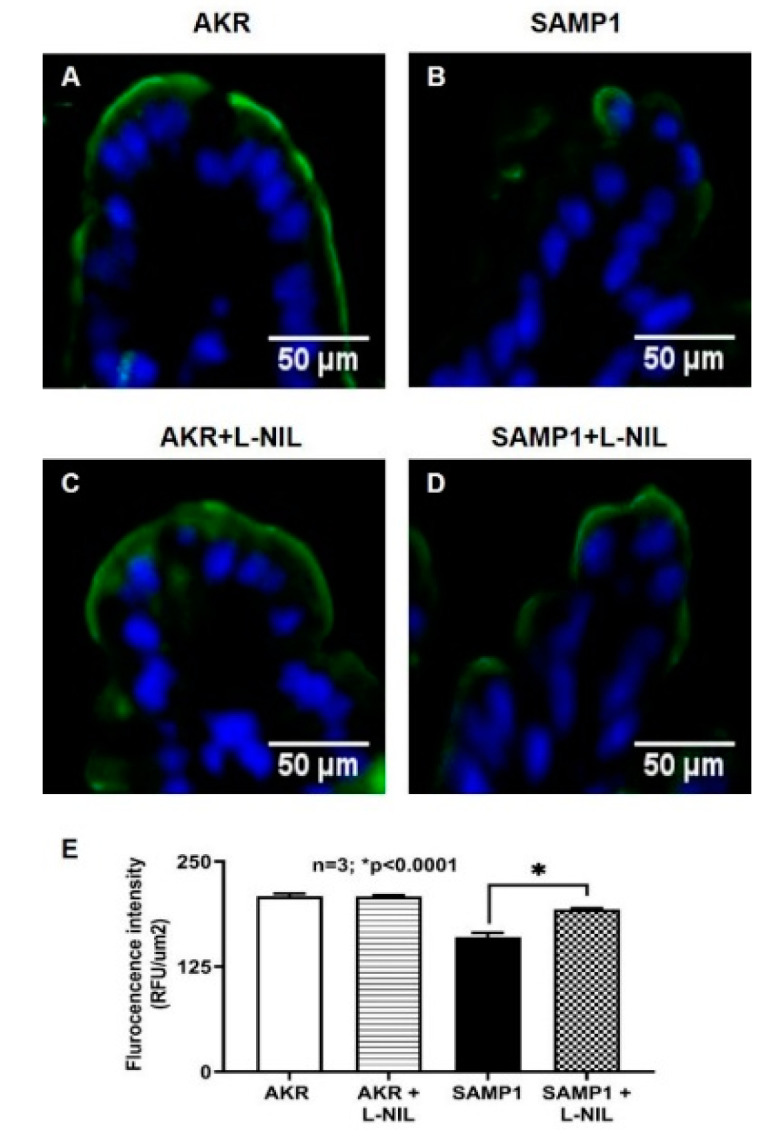
Immunofluorescence of SGLT1 in SAMP1 mice intestine. Images captured at 10X magnification. (**A**) SGLT1 in AKR mouse intestine, demonstrating its abundance in the BBM of villus cells lining the villus. (**B**) SAMP1 mouse intestine demonstrating the diminished SGLT1 in the BBM of villus cells lining the villus. (**C**) SGLT1 in L-NIL treated AKR mouse intestine, demonstrating that SGLT1 expression is unchanged in the BBM of villus cells. (**D**) L-NIL treated SAMP1 mouse intestine demonstrating the restoration of diminished SGLT1 in the BBM of villus cells. (**E**) Quantitation of SGLT1 in AKR and SAMP1 mice intestine, demonstrating that L-NIL treatment reverses the inhibition of SGLT1 in SAMP1 mice.

To further confirm the molecular mechanism of inhibition of SGLT1 protein expression, immunofluorescence studies were performed. Images were captured at 10X magnification. Figure 10A shows SGLT1 in AKR mice intestine, demonstrating its abundance in the BBM of villus cells lining the villus. However, as shown in Figure 10B, SGLT1 protein expression was markedly decreased in SAMP1 mice ileal villus cells. But, L-NIL treatment reversed this decrease to normal levels comparable to that from AKR mice (Figure 10D). Moreover, L-NIL treatment did not change SGLT1 protein levels in AKR mice villus cells (Figure 10C). The quantitation of SGLT1 fluorescence levels confirmed these findings (Figure 10E).

## 4. Discussion

This study demonstrated that SGLT1 activity is significantly decreased in villus cells from SAMP1 mouse model of chronic ileitis. Further, iNO production was increased in villus cells in SAMP1 mice and inhibition of it reversed SGLT1 inhibition to its normal levels comparable to control AKR mice. In the intact villus cells, inhibition of SGLT1 activity by iNO was secondary to both an effect at the level of the BLM Na^+^/K^+^-ATPase as well as BBM SGLT1 co-transporter. At the level of the co-transporter in the BBM, the mechanism of inhibition of SGLT1 was secondary to reduced BBM co-transporter numbers. Finally, the mechanism of L-NIL mediated restoration of decreased Na-glucose co-transport activity in ileal villus cells was by restoring the number of BBM SGLT1 protein to its normal levels.

IBD, consisting of Crohn’s disease and ulcerative colitis, afflicts more than 3.5 million patients in the Western hemisphere with increasing incidence over the last five decades [3]. IBD is characterized by chronic, unrelenting intestinal inflammation, villus blunting and crypt hypertrophy that leads to malabsorption of fluid, electrolytes and nutrients as well as secretion of fluid and electrolytes [28,29,30,31,32]. This in IBD results in its most common and disabling symptoms, diarrhea, malnutrition, weight loss and in the pediatric population, a failure to thrive. It is fairly well understood that IBD occurs in the genetically susceptible host, when environmental factors perturb the intestinal microbiome, resulting in an immune response which is excessive, dysregulated and alters intestinal absorption and secretion. Many immune-inflammatory mediators have been shown to be elevated in the inflamed mucosa that may affect intestinal electrolyte and nutrient transport [32,33,34,35]. Of the nutrients, glucose is the most abundant one in the human diet [2]. It is primarily absorbed in the mammalian small intestine by a secondary active transport process called Na-glucose co-transporter 1 (SGLT1; SLC5A1) located on the brush border membrane (BBM) of the absorptive villus, but not secretory crypt cells. The necessary Na-gradient for this BBM co-transporter is provided by Na^+^/K^+^-ATPase on the basolateral membrane (BLM) of enterocytes. Thus, the regulation of SGLT1 activity may be at the level of the co-transporter and/or at Na^+^/K^+^-ATPase. However, how SGLT1 may be regulated in the IBD intestine and specifically by one of the most biologically active molecules, NO, known to be elevated in the IBD mucosa was not previously known.

In this mouse model of IBD, SGLT1 is diminished in intact villus cells. The inhibition is secondary to a decrease in the favorable Na-gradient due to altered activity of the BLM Na^+^/K^+^-ATPase as well as an effect at the level of the co-transporter in the BBM as reflected by the decreased Na-dependent glucose uptake in BBMV studies. RT-qPCR study revealed that SGLT1 mRNA expression levels were significantly reduced in SAMP1 mice model of ileitis. Western blot studies demonstrated that SGLT1 protein was decreased in the whole cell homogenates from the chronically inflamed ileum. Further, BBM Western blot studies demonstrated that the molecular mechanism of inhibition of SGLT1 during chronic ileitis is likely secondary to the reduced synthesis of SGLT1 as compared to altered trafficking to the BBM. Finally, immunofluorescence studies confirmed these findings.

Previously, in a rabbit model of chronic intestinal inflammation resembling IBD and featuring specific alterations in electrolyte and nutrient transport pathways, it was demonstrated that SGLT1 was inhibited [24,36]. In the chronically inflamed rabbit ileum, it was shown that although the activity of Na^+^/K^+^-ATPase was diminished, it was not the sole explanation for the decrease in SGLT1 [24]. In fact, SGLT1 was inhibited at the level of the co-transporter in the BBM as a result of a decrease in the number of co-transporters without a change in the affinity for glucose. Thus, the observations pertaining to SGLT1 in the SAMP1 mouse model of IBD are comparable to the rabbit model of IBD. However, it was not known whether iNO mediates the changes in SGLT1 in vivo in the chronically inflamed intestine in SAMP1 mouse.

In prior studies, iNO has been shown to affect the changes in glutamine assimilation in the chronically inflamed rabbit intestine [37]. Glutamine is the primary metabolic fuel for intestinal enterocytes. It is now considered a “conditionally essential” amino acid as compared to the prior paradigm of a non-essential amino acid [38]. Glutamine is assimilated in villus cells through a sodium dependent co-transport process in the BBM specifically via B0AT1. In the rabbit model of chronic intestinal inflammation, B0AT1 was inhibited in villus cells secondary to a decrease in the BBM co-transporter numbers. Treatment of chronic enteritis rabbits in vivo with L-NIL reversed the inhibition of B0AT1. The mechanism of reversal of B0AT1 activity by L-NIL was by restoring BBM co-transporter numbers [37].

In the present study, NO levels are increased in the SAMP1 mice enterocytes. Treatment of mice in vivo with L-NIL reduced the NO levels to normal. It also reversed the inhibition of SGLT1 in villus cells. At the cellular level, the diminished Na gradient was restored by L-NIL. At the level of the co-transporter in the BBM, both the activity as well as the co-transporter numbers was restored by L-NIL treatment. Thus, the exact mechanisms by which SGLT1 was inhibited in the chronically inflamed intestine are reversed when elevated NO levels are reduced to normal levels in the SAMP1 mice. In the control AKR mice, L-NIL had no effect on NO, Na^+^/K^+^-ATPase or SGLT1. These results indicate that iNO likely mediates the alteration in SGLT1 in the chronically inflamed intestine of SAMP1 mice.

NO is known to regulate transporter proteins via cGMP mediated Protein Kinase G pathway or through direct nitrosylation [39,40]. In the SAMP1 villus cells, iNO likely inhibits SGLT1 by downregulating SGLT1 mRNA transcription through modulation of SGLT1 gene specific transcription factors, since SGLT1 activity has been shown to be altered through changes in co-transporter numbers. NO could also mediate its effects by first forming peroxynitrite (OONO), a potent oxidant. In fact, iNO and superoxide levels are known to be increased in the inflamed mucosa, where they combine together to produce an abundant amount of OONO. OONO has been shown in vitro in rat intestinal epithelial cells (IEC-18 cells) to inhibit SGLT1 [41]. In this study, the OONO mediated mechanism of inhibition of SGLT1 was secondary to a significant decrease in enterocyte BBM SGLT1 protein expressions. Further, this study demonstrated that the p38 mitogen-activated protein (MAP) kinase pathway likely mediated the OONO inhibition of SGLT1. Finally, at the level of the SGLT1 co-transporter, 3-nitrotyrosine formation appeared to be the mechanism of inhibition of SGLT1 in IEC-18 cells [41].

## 5. Conclusions

In the SAMP1 mouse model of IBD, Na-glucose co-transport is inhibited in villus cells. At the cellular level, both an effect at the level of Na extrusion capacity of the cell as well as an effect at the co-transporter mediates the downregulation of SGLT1. At the level of the co-transporter in the BBM of villus cells, the mechanism of inhibition during chronic enteritis is secondary to a reduction in the synthesis of SGLT1 protein. This inhibition of SGLT1 in SAMP1 mouse intestine appears to be mediated by iNO. Reversal of the increase in iNO in the SAMP1 mouse intestine restores SGLT1 activity by restoring both the Na-gradient of the villus cells as well as by reversing the reduced synthesis of SGLT1 protein. Thus, inducible nitric oxide uniquely regulates glucose assimilation in the chronically inflamed intestine.
